# Asthma in Pregnancy: A Critical Review of Impact, Management, and Outcomes

**DOI:** 10.7759/cureus.50094

**Published:** 2023-12-07

**Authors:** Anisha P Vyawahare, Abhay Gaidhane, Bhushan Wandile

**Affiliations:** 1 Medicine, Jawaharlal Nehru Medical College, Datta Meghe Institute of Higher Education & Research, Wardha, IND; 2 Public Health, Jawaharlal Nehru Medical College, Datta Meghe Institute of Higher Education & Research, Wardha, IND; 3 Hospital Administration, Jawaharlal Nehru Medical College, Datta Meghe Institute of Medical Science, Wardha, IND

**Keywords:** asthma management, precision medicine, medication safety, fetal outcomes, maternal health, asthma in pregnancy

## Abstract

Asthma is a common chronic respiratory condition that can significantly impact the health of pregnant women and their developing fetuses. This comprehensive review provides insights into the prevalence of asthma in pregnant women, the physiological changes during pregnancy, and the multifaceted impact of asthma on maternal and fetal health. It emphasizes the importance of proper asthma diagnosis, medication management, and the development of personalized asthma action plans during pregnancy. Lifestyle modifications, trigger avoidance, and stress reduction are essential to effective management. Healthcare providers are pivotal in educating, monitoring, and individualized care to ensure optimal asthma control. The review underscores the critical significance of managing asthma during pregnancy, as it improves maternal and fetal outcomes and potentially influences long-term health for both mother and child. Future directions in this field involve ongoing research, personalized treatment, early intervention, and precision medicine to enhance the understanding and care of asthma during pregnancy.

## Introduction and background

Asthma is a chronic respiratory condition characterized by airway inflammation and hyperresponsiveness, resulting in recurring breathlessness, coughing, and wheezing episodes. It affects individuals of all ages, races, and genders and is a significant public health concern. The prevalence of asthma has been steadily increasing in recent decades, with millions of people worldwide living with this condition. However, one unique and critical aspect of asthma is its interaction with pregnancy [1]. Asthma is a condition that affects the airways, leading to periods of bronchoconstriction and inflammation. Common triggers for asthma symptoms include allergens, viral infections, irritants, and exercise. These episodes can be mild, moderate, or severe and life-threatening in severe cases. According to the World Health Organization (WHO), an estimated 235 million people worldwide have asthma, a leading cause of chronic disease [2].

Pregnancy is a unique physiological state during which significant changes occur in a woman's body to support fetal development. These changes can profoundly impact a woman's health and respiratory system. Asthma management and its effects on maternal and fetal health become crucial considerations during this period [3]. The importance of studying asthma in pregnancy cannot be overstated. Pregnant women with asthma require special care and attention because poorly controlled asthma can lead to adverse outcomes for both the mother and the baby. Understanding the impact of asthma during pregnancy and developing effective strategies for its management is essential for ensuring the well-being of both the expectant mother and the developing fetus [4]. This comprehensive review aims to provide an in-depth analysis of asthma's impact, management, and outcomes in pregnancy. By delving into the complex interplay between asthma and pregnancy.

## Review

Prevalence of asthma in pregnant women

Asthma is a prevalent chronic respiratory condition that affects individuals of all ages, including women of childbearing age. Among pregnant women, bronchial asthma (BA) is the most common chronic disease, complicating approximately 4% to 8% of pregnancies. Understanding the prevalence of asthma in pregnant women is a fundamental aspect of recognizing the significance of this health concern during pregnancy. Notably, asthma can manifest for the first time during pregnancy, or women with pre-existing asthma may experience changes in their symptoms and management strategies [4]. Epidemiological studies have consistently demonstrated the common occurrence of asthma among pregnant women, with prevalence rates ranging from 3% to 8% across various populations. This positions asthma as one of the most prevalent chronic conditions affecting expectant mothers. The prevalence of asthma during pregnancy is influenced by a complex interplay of factors, including genetic predisposition, environmental exposures, and hormonal changes. Furthermore, disparities in asthma prevalence among different racial and socioeconomic groups underscore the importance of addressing health equity in asthma care during pregnancy [5].

Physiological changes during pregnancy

Hormonal Changes

During pregnancy, there is a significant surge in certain hormones, notably estrogen and progesterone. These hormones have a broad-reaching impact on the body, including the respiratory system. Estrogen, for example, can affect airway smooth muscle tone and reactivity. This hormonal influence can be a double-edged sword for pregnant women with asthma. While it can alleviate symptoms for some, it may exacerbate asthma for others. Understanding these hormonal dynamics is pivotal as it sheds light on why asthma symptoms vary among pregnant women and underscores the importance of personalized asthma management [6].

Cardiovascular Changes

The cardiovascular system undergoes substantial adaptations during pregnancy. Increased cardiac output ensures an adequate supply of oxygen and nutrients to the developing fetus. This results in changes in pulmonary circulation to facilitate oxygen exchange. While these cardiovascular adaptations are essential for the growing baby's well-being, they can also impact the respiratory system. Pregnant women may experience altered breathing patterns, increased shortness of breath, and changes in their perception of asthma symptoms. These intricate interactions emphasize the need for healthcare providers to consider the broader physiological context when managing asthma in pregnancy [7].

Mechanical changes: As the fetus grows, it occupies space within the abdominal cavity. This physical change leads to shifts in the position of the diaphragm and ribcage. For women with asthma, these mechanical alterations can significantly affect lung function. Altered diaphragmatic and ribcage positions may affect the volume of air a pregnant woman can inhale and exhale, potentially exacerbating asthma-related breathing difficulties. The awareness of these mechanical changes is essential in developing tailored asthma management plans that consider the evolving anatomy of the pregnant woman [8].

Impact of asthma on maternal and fetal health

Maternal Health

Recognizing the profound impact of poorly controlled asthma on maternal health during pregnancy is paramount. When asthma is not effectively managed, expectant mothers may face a higher risk of exacerbations, an increased burden of asthma symptoms, and a notable decrease in their overall quality of life. In severe instances, uncontrolled asthma can lead to respiratory distress, necessitating hospitalization, and, in extreme cases, may even culminate in life-threatening asthma attacks. Thus, the consequences of inadequate asthma management for maternal well-being are far-reaching and demand diligent attention and care [9].

Fetal Health

Equally significant is the understanding that the ramifications of maternal asthma can extend to the developing fetus. A mounting body of research underscores the association between uncontrolled asthma during pregnancy and a heightened risk of adverse fetal outcomes. These outcomes encompass preterm birth, where babies are delivered before completing a full term, low birth weight, which can predispose newborns to various health challenges, and an elevated likelihood of congenital anomalies. Furthermore, there is growing evidence that maternal asthma may exert a lasting influence on the offspring's health, potentially increasing the risk of childhood asthma and allergic diseases. The impact of maternal asthma thus reverberates into the next generation, emphasizing the imperative need for effective asthma management during pregnancy to safeguard maternal and fetal health [10].

Impact of asthma on pregnancy

Exacerbation Risk and Severity

Asthma exacerbations during pregnancy represent a critical concern for maternal health, demanding a thorough understanding of the risk and severity involved. Exacerbations are characterized by the sudden and pronounced worsening of asthma symptoms, often necessitating urgent medical attention. Such comprehension is vital for the well-being of pregnant women dealing with asthma [11]. Pregnancy is recognized as a potential risk factor for asthma exacerbations, with the second and third trimesters being particularly vulnerable. Hormonal fluctuations, increased blood volume, and alterations in lung mechanics collectively contribute to an elevated risk of exacerbations in pregnant women with asthma [5]. Severe exacerbations have the potential to precipitate respiratory distress and, in some instances, require hospitalization. These severe episodes directly threaten the mother's health, carrying the added burden of impacting fetal well-being. It is imperative to identify the contributing factors to the severity of exacerbations to devise effective prevention and management strategies [12].

Impact on Maternal Quality of Life

Asthma during pregnancy can substantially impact expectant mothers' overall quality of life. Poorly controlled asthma symptoms, the constant apprehension of exacerbations, and concerns regarding the safety of asthma medications can collectively influence various facets of maternal well-being [13]. Frequent asthma symptoms and exacerbations can inflict physical discomfort and emotional distress on expectant mothers. Maternal anxiety and stress related to asthma may have broader repercussions on pregnancy outcomes, underscoring the need for holistic support and care [14]. Many pregnant women with asthma grapple with concerns about the safety of asthma medications during pregnancy. The delicate balance between the imperative need for adequate symptom control and the potential risks associated with medication use can be a source of considerable distress, significantly impacting maternal well-being. Comprehensive guidance and support in navigating these concerns are essential to ensure the health and quality of life of pregnant women with asthma [15].

Fetal health

Potential Effects on Fetal Development

Asthma in pregnancy carries the potential to influence fetal development through various mechanisms, warranting a careful examination of its impact on the developing fetus [5]. The inflammation and respiratory distress associated with maternal asthma may affect placental function. The impaired placental function can potentially diminish the supply of oxygen and nutrients to the fetus, affecting growth and overall development [16]. Maternal asthma can trigger inflammatory responses that hold the capacity to impact the developing fetal immune system. The precise implications of these responses on the child's long-term health remain an active area of research, highlighting the intricacies of the mother-fetal interplay during pregnancy [17].

Risk of Preterm Birth and Low Birth Weight

Preterm birth and low birth weight are consequential complications often associated with maternal asthma, with significant implications for the newborn's health [18]. Pregnant women grappling with uncontrolled asthma face an elevated risk of preterm birth. This, in turn, is linked to an increased likelihood of neonatal complications and enduring health challenges for the child, underscoring the urgency of effective asthma management during pregnancy [18]. Poorly controlled asthma throughout pregnancy can contribute to low birth weight, further elevating the risk of health issues for the newborn. Understanding the underlying factors leading to these outcomes is paramount, forming the basis for prevention and intervention strategies [11].

Congenital Anomalies and Childhood Asthma Risk

The potential association between maternal asthma and congenital anomalies in the developing fetus remains a subject of ongoing research. Evidence suggests that pregnancy-related asthma may influence the offspring's childhood asthma and allergic disease risk [19]. Preliminary studies have hinted at a potential link between maternal asthma and an increased risk of congenital anomalies in newborns. Nevertheless, further research is essential to clarify the nature and nuances of this relationship, offering a more comprehensive understanding of its implications [20]. Children born to mothers with asthma may encounter an escalated risk of developing asthma and associated allergic conditions. Exploring the mechanisms underpinning this association and identifying potential preventive measures is critical in optimizing the offspring's long-term health. The intricate interplay between maternal asthma and its implications for fetal development and childhood health emphasizes the significance of astute management and research in this field [18].

Diagnosis and monitoring

Methods for Diagnosing Asthma in Pregnant Women

Clinical assessment: The diagnostic journey for asthma often commences with a comprehensive clinical assessment. This crucial step entails healthcare providers conducting a detailed review of the patient's medical history, paying particular attention to any previous history of asthma, other respiratory conditions, allergies, and asthma-related symptoms. Asthma symptoms, such as wheezing, coughing, chest tightness, and shortness of breath, are meticulously explored during this assessment. This comprehensive overview allows healthcare providers to gain essential insights into the patient's respiratory health [21].

Pulmonary function testing: Pulmonary function testing, frequently employing spirometry, is a pivotal diagnostic tool for asthma. Spirometry measures critical lung function parameters, including forced expiratory volume in one second (FEV1) and forced vital capacity (FVC). These measurements prove invaluable in confirming the presence of obstructive airway disease, offering objective data to support the diagnostic process. This quantitative assessment plays a central role in distinguishing asthma from other respiratory conditions [22].

Peak expiratory flow (PEF) measurement: Portable peak flow meters are essential for measuring PEF. Over time, monitoring changes in PEF can offer critical insights into asthma control. Moreover, it aids in diagnosing asthma, serving as a dynamic tool that provides real-time information about airway function and responsiveness. PEF measurement is especially advantageous in longitudinal asthma management, enabling healthcare providers to track trends and make informed treatment decisions [23].

Bronchial challenge tests: Bronchial challenge tests, such as those involving methacholine or exercise challenges, are conducted to induce bronchoconstriction within a controlled setting. These tests hold significant diagnostic value as they serve to confirm the presence of asthma and assess airway hyperresponsiveness. By exposing the airways to specific challenges, healthcare providers can gauge the degree of bronchoconstriction, a hallmark feature of asthma, thereby enhancing diagnostic accuracy [24].

Allergy testing: In cases where allergic asthma is suspected, allergy testing is integral to the diagnostic process. Allergy skin tests or blood tests measuring serum IgE levels are employed to identify specific allergens that could trigger asthma symptoms. Healthcare providers can confirm the diagnosis and tailor treatment strategies to address the underlying triggers by pinpointing these allergens. Allergy testing is a vital component of the diagnostic approach, particularly in cases where allergies contribute to asthma symptoms [25].

Importance of Accurate Diagnosis

Tailored treatment: The cornerstone of accurate asthma diagnosis lies in the ability to create a personalized asthma management plan that considers the unique intricacies of the condition, specific triggers, and the individual requirements of the pregnant woman. Tailored treatment is not just a preference but an essential component in optimizing symptom control while mitigating potential risks to the developing fetus. This precision in care ensures that expectant mothers receive treatment strategies that are both effective and safe, catering to their specific needs and health dynamics [26].

Safety considerations: An accurate diagnosis has significant implications for ensuring the safety of the pregnant woman and her developing fetus. It paves the way for healthcare providers to make well-informed decisions regarding prescribing asthma medications. The safety of these medications during pregnancy varies, and an accurate diagnosis is critical in guiding healthcare providers in selecting the most appropriate asthma medications. This delicate balance between symptom control and fetal safety underscores the importance of precision in diagnosis, safeguarding the well-being of both mother and child [15].

Prevention of complications: One of the most pivotal roles of accurate diagnosis is preventing complications associated with maternal asthma. By pinpointing the exact nature and severity of the condition, timely interventions can be implemented to prevent complications such as asthma exacerbations, preterm birth, and low birth weight. Ensuring optimal asthma control becomes a primary goal of diagnosis, reducing the risks of adverse outcomes for both the mother and the developing fetus. Accurate diagnosis thus is a linchpin in comprehensive asthma care during pregnancy, potentially enhancing maternal and fetal health outcomes [27].

Monitoring Asthma Symptoms and Lung Function During Pregnancy

Regular follow-up: Pregnant women managing asthma should prioritize regular follow-up appointments with their healthcare providers. These scheduled visits play a vital role in the ongoing assessment of asthma control. During these appointments, healthcare providers can evaluate the patient's asthma status, make necessary medication adjustments, and closely monitor any changes in symptoms. The consistent follow-up ensures that evolving needs or concerns are addressed promptly, contributing to optimal asthma management during pregnancy maintenance [9].

Symptom diary: Maintaining a detailed symptom diary is invaluable in proactive asthma management. Pregnant women are encouraged to meticulously record their asthma symptoms, documenting the frequency and severity of occurrences, potential triggers, and medication use. This diary is a crucial aid for the patient and healthcare providers. It facilitates the ongoing assessment of asthma control, helping to identify patterns and trends in symptom fluctuations and enabling appropriate adjustments to the treatment plan. Systematic record-keeping empowers pregnant women with asthma to actively engage in their care [9].

Lung function testing: Regular pulmonary function testing, encompassing tests like spirometry and PEF measurements, is a cornerstone of the monitoring process for pregnant women with asthma. These tests provide objective data on lung function and capacity. Changes in lung function can serve as early indicators of deteriorating asthma control. Consistent lung function testing offers a dynamic and quantitative means of assessing the efficacy of the current treatment regimen and guiding healthcare providers in making timely adjustments to maintain asthma control [28].

Asthma action plan: Developing and utilizing a personalized asthma action plan is paramount for pregnant women. This plan outlines a systematic approach for the patient to follow in response to worsening symptoms or exacerbations. It is a valuable resource for patients to understand the steps to take when asthma control falters and empowers them to seek prompt medical attention when needed. The asthma action plan provides a clear and structured framework for managing asthma exacerbations and is essential to asthma self-management during pregnancy [9].

Management of asthma in pregnancy

Medications

Medications play a crucial role in effective asthma management during pregnancy, requiring careful consideration of the safety and efficacy of various medication options to ensure the well-being of both the mother and the developing fetus. Central to this process is optimizing symptom control while minimizing potential risks. First, regarding the safety of asthma medications during pregnancy, inhaled corticosteroids are typically considered safe as they are fundamental in controlling asthma and mitigating the risks associated with uncontrolled asthma, which can have more adverse consequences [26]. Although short-acting and long-acting bronchodilators, including beta-agonists, are generally deemed safe during pregnancy, healthcare providers must carefully assess the most suitable option based on individual needs. While leukotriene modifiers like montelukast are occasionally prescribed for asthma management, their safety during pregnancy is still under investigation, necessitating careful consideration of potential risks and benefits. Systemic corticosteroids are generally avoided during pregnancy due to their potential adverse effects. Still, in severe cases, the benefits may outweigh the risks, prompting healthcare providers to make well-informed decisions based on careful evaluation [29].

Moreover, adjusting medication doses is critical to managing asthma during pregnancy. An individualized approach to medication dosages is essential to balance achieving optimal asthma control and minimizing potential risks to the fetus. Considering the physiological changes throughout pregnancy, pregnant women may require dose adjustments as their pregnancy progresses. Regular follow-up appointments with healthcare providers are vital to monitor the need for such adjustments [26]. Healthcare providers will closely monitor asthma control and make necessary changes to medication regimens, ensuring that the treatment plan remains effective and safe. This proactive approach, coupled with regular monitoring and adjustment, is pivotal in ensuring that pregnant women receive the appropriate asthma management tailored to their specific needs, contributing to a safer and healthier pregnancy for both the mother and the developing fetus [30].

Asthma Action Plan

Creating and implementing a personalized asthma action plan for pregnant women is essential for effectively managing their condition and ensuring the safety of both the mother and the developing fetus. Such a plan is tailored to each individual's needs and circumstances, considering their unique triggers and medication requirements. This personalized approach to asthma management is crucial for several reasons. Firstly, a well-crafted action plan provides pregnant women with clear guidelines and instructions on responding to changes in their asthma symptoms or exacerbations [31]. By outlining specific steps to take during these instances, the plan empowers women to take control of their condition and make informed decisions about their health. This includes understanding when to seek immediate medical attention and when to adjust their medications, enabling them to effectively manage their asthma and take prompt action during worsening symptoms [32].

Furthermore, patient education is pivotal in effectively empowering pregnant women to manage their asthma. Comprehensive education should encompass various aspects of the condition, including its potential impact on pregnancy, the proper use of medications, and the importance of adhering to a personalized action plan. By understanding the risks and benefits of different treatment options, pregnant women can make informed decisions about their health and actively participate in their asthma management. Regular follow-up appointments with healthcare providers are integral to this process, allowing for the continuous assessment of asthma control, the timely address of any concerns, and the necessary adjustment of the treatment plan as required. Adhering to these follow-up appointments is crucial to ensure that any changes in the asthma management plan are promptly implemented, promoting effective symptom control and maintaining the overall well-being of the pregnant woman and the developing fetus. By emphasizing the importance of a personalized action plan, patient education, and adherence to follow-up appointments, healthcare providers can contribute to a safer and healthier pregnancy experience for women with asthma [29].

Lifestyle and Environmental Factors

Minimizing exposure to triggers is a fundamental aspect of managing asthma during pregnancy. This preventive approach aims to reduce the risk of asthma exacerbations and maintain optimal respiratory health for both the mother and the developing fetus [30]. Firstly, allergen control is critical in managing asthma triggers. Identifying and avoiding specific allergens that can exacerbate asthma symptoms is crucial. Common allergens, such as dust mites, pollen, and pet dander, can significantly impact asthma control. Pregnant women should take proactive measures to minimize exposure to these triggers in their home environment. This may involve regular cleaning to reduce dust and allergens, using allergen-proof covers on mattresses and pillows, and keeping pets away from living areas to mitigate the risk of triggering asthma symptoms [33].

Additionally, avoiding exposure to environmental tobacco and second-hand smoke is vital for pregnant women with asthma. Second-hand smoke can significantly worsen asthma symptoms and pose severe maternal and fetal health risks. Pregnant women must avoid environments where they might be exposed to second-hand smoke, whether at home or in public places [34]. For pregnant women who smoke, quitting smoking is of utmost importance. Smoking is a known risk factor for compromised asthma control, and it can contribute to preterm birth and low birth weight. Healthcare providers should prioritize providing support and resources to help pregnant women quit smoking. By facilitating smoking cessation, healthcare professionals can contribute to improved asthma control and better overall health outcomes for both the mother and the developing fetus [35].

Asthma control and maternal outcomes

Strategies for Maintaining Asthma Control During Pregnancy

Medication adherence: Ensuring strict adherence to prescribed medication regimens is a cornerstone of effective asthma management for pregnant women. This commitment to consistent medication use, especially with controller medications like inhaled corticosteroids, plays a pivotal role in asthma control and the prevention of exacerbations. The timely and regular use of these medications aligns with healthcare providers' recommendations and is a fundamental step in safeguarding maternal and fetal well-being during pregnancy [36].

Asthma action plan: Diligent adherence to a personalized asthma action plan is indispensable for pregnant women with asthma. A comprehensive understanding of when and how to adjust medications, when to seek medical attention, and the steps to take during various asthma-related scenarios is paramount. These action plans are customized to each patient's needs and offer a structured framework for responding to fluctuating asthma symptoms or exacerbations, empowering pregnant women to take proactive and informed measures to preserve asthma control [37].

Regular monitoring: Consistent monitoring of asthma symptoms and lung function is a non-negotiable aspect of asthma management during pregnancy. Pregnant women should actively self-monitor by tracking their PEF and keeping a detailed symptom diary. These tools provide valuable insights into symptom patterns and trends, enabling timely interventions. Furthermore, regular follow-up appointments with healthcare providers serve as essential check-ins to assess asthma control. These appointments include lung function tests and symptom evaluations, which guide treatment plan adjustments and ensure effective asthma management [5].

Allergen and trigger control: Identifying and mitigating asthma triggers is crucial for pregnant women. This process may involve environmental changes to reduce exposure to allergens and irritants, thereby minimizing the risk of asthma exacerbations. By taking proactive measures to control potential triggers, expectant mothers can contribute to maintaining stable asthma control and mitigating symptom flare-ups [38].

Stress reduction: Managing stress and anxiety can positively impact asthma control during pregnancy. Stress-reduction techniques, such as mindfulness, relaxation exercises, and prenatal yoga, can be valuable tools. By incorporating stress-reduction practices into their routine, pregnant women can create a more supportive environment for asthma management, ultimately benefiting their physical and emotional well-being while ensuring maternal and fetal health are optimized during this dynamic period [39].

Effect of Asthma Control on Maternal Outcomes

Improved quality of life: Achieving and maintaining adequate asthma control during pregnancy yields substantial benefits by significantly enhancing expectant mothers' overall quality of life. Women who experience well-controlled asthma typically encounter fewer asthma symptoms, less frequent exacerbations, and reduced anxiety and stress levels related to their condition. These improvements in symptom burden and psychological well-being contribute to a more positive and comfortable pregnancy experience. Pregnant women can engage more actively in their daily routines, enjoy a greater sense of normalcy, and experience reduced disruptions to their daily activities, ultimately resulting in an improved overall quality of life [40].

Reduced risk of exacerbations: Effective asthma management translates to a decreased risk of asthma exacerbations during pregnancy, which carries several profound advantages. Lowering the risk of exacerbations alleviates the physical and emotional burden on expectant mothers and minimizes the need for hospitalization. Hospitalization for severe asthma exacerbations can be particularly concerning during pregnancy, as it may introduce additional complexities and stress. Moreover, by preventing exacerbations, the likelihood of respiratory distress and related complications is significantly diminished, reducing the potential adverse consequences for both the mother and the developing fetus. Reducing exacerbation risk is pivotal to optimizing maternal and fetal well-being during pregnancy [41].

Optimal fetal health: Maternal asthma control indirectly yet vitally affects fetal health. When a pregnant woman maintains consistent asthma control, she provides a more stable and supportive environment for the developing fetus. This environment minimizes the risk of preterm birth and low birth weight, which, in turn, has positive implications for both the mother and child. Preterm birth and low birth weight are significant risk factors for neonatal complications and long-term health issues for the child. By optimizing fetal health through maternal asthma control, expectant mothers can enhance the prospects of a healthier and smoother pregnancy and improve their child's long-term well-being, aligning with their aspirations for a positive and fulfilling maternal experience [42].

The Role of Healthcare Providers in Optimizing Asthma Control

Comprehensive assessment: The cornerstone of effective asthma management during pregnancy lies in conducting a comprehensive assessment of the pregnant woman's asthma. This process entails thoroughly evaluating her asthma history, including any previous diagnoses and treatment regimens, current symptoms, and the severity of her asthma. This in-depth assessment is the foundation for informed treatment decisions, ensuring that asthma management is not a one-size-fits-all approach. Instead, it is tailored to the individual's unique needs and circumstances, aligning the treatment plan with the specific nuances of her condition [43].

Individualized treatment plans: Tailored treatment plans are paramount in addressing asthma during pregnancy. These plans are meticulously developed for each expectant mother, considering many factors. They account for the safety of asthma medications during pregnancy, the severity of her asthma, potential triggers, and any specific concerns or preferences she may have. This individualized approach fosters collaboration between healthcare providers and the patient, empowering her to make informed choices about asthma management. Considering the nuances of her condition, her treatment plan is effective and safe for her and her developing fetus [26].

Patient education: One of the pillars of successful asthma management is comprehensive patient education. Healthcare providers are pivotal in conveying essential information about asthma to pregnant women. This education covers various topics, including the significance of adhering to prescribed medications, the correct inhaler techniques, and the ability to recognize and respond to asthma symptoms. By providing this knowledge, healthcare providers empower pregnant women to take an active role in managing their condition effectively, fostering a sense of control and confidence in their ability to navigate their asthma during pregnancy [44].

Regular follow-up: Regular follow-up appointments are essential in ensuring ongoing asthma control and making necessary adjustments to the treatment plan as circumstances evolve. These appointments are vital for healthcare providers to monitor the patient's lung function, gauge symptom control, and assess adherence to the action plan. The insights gained during these visits guide any needed adaptations to the treatment regimen, ensuring that asthma management remains optimized for the duration of the pregnancy [45].

Collaboration: Effective asthma management during pregnancy requires seamless collaboration among healthcare team members. This includes asthma specialists, obstetricians, and neonatologists, who may become involved in addressing potential complications due to asthma during pregnancy. This interdisciplinary approach to care ensures that all facets of the patient's health are considered, promoting coordinated and holistic management and prioritizing maternal and fetal well-being. The collaborative efforts of the healthcare team are essential in addressing any complexities or challenges that may emerge, working collectively to provide the highest standard of care for expectant mothers with asthma [46].

Asthma control and fetal outcomes

Strategies for Improving Fetal Outcomes in Pregnant Women With Asthma

Optimal asthma control: The cornerstone of enhancing fetal outcomes during pregnancy revolves around maintaining optimal asthma control. When a pregnant woman effectively manages her asthma, her respiratory system functions efficiently, guaranteeing a consistent and adequate supply of oxygen and essential nutrients to the developing fetus. This controlled environment nurtures a healthier developmental landscape, reducing the potential risks associated with inadequate oxygenation and nutrient delivery. Thus, focusing on asthma control is a fundamental strategy to support fetal well-being and overall pregnancy health [29].

Medication safety: A pivotal aspect of optimizing fetal outcomes in pregnant women with asthma is the judicious selection of asthma medications. Ensuring that these medications are considered safe during pregnancy is paramount. Collaborating closely with healthcare providers to identify the most appropriate medications and establish tailored dosage regimens becomes essential. This fine-tuned approach is aimed at balancing the imperative of symptom control with the critical objective of ensuring fetal safety. By making informed choices about medication selection and dosage, expectant mothers can navigate their asthma management effectively, contributing to positive fetal outcomes [15].

Lifestyle modifications: Advancing overall maternal health through lifestyle modifications is a crucial strategy. Encouraging pregnant women to adopt and sustain balanced dietary habits, engage in regular, safe physical activity, and maintain a healthy weight becomes pivotal in supporting fetal well-being. These modifications foster a nurturing and supportive environment for the developing fetus, offering benefits beyond asthma management to comprehensively nurture the overall health of both mother and child [47].

Stress reduction: Employing effective stress management techniques emerges as an additional strategy to enhance fetal outcomes. Techniques such as mindfulness, relaxation exercises, and counseling can significantly reduce maternal stress, indirectly benefiting fetal health. By mitigating stressors, expectant mothers create a more harmonious and stable intrauterine environment, reducing the potential impact of maternal stress on fetal development [48].

Nutritional support: Ensuring that pregnant women receive an adequate intake of essential nutrients and vitamins is fundamental for fetal development. Healthcare providers may recommend incorporating prenatal vitamins to meet specific nutritional needs. By addressing these nutritional requirements, the developmental trajectory of the fetus is positively influenced, setting the stage for healthy growth and overall well-being [49].

Smoking cessation: Smoking cessation should be a top priority if the pregnant woman is a smoker. This imperative action aims to prevent the adverse effects of tobacco smoke exposure on fetal health. Smoking during pregnancy can lead to many complications, including low birth weight, preterm birth, and developmental issues. By prioritizing smoking cessation, expectant mothers take a proactive step toward safeguarding the health and well-being of their developing child, aligning to foster positive fetal outcomes during pregnancy [50].

The Impact of Asthma Control on Fetal Outcomes

Optimized fetal growth: One of the primary objectives of well-controlled asthma during pregnancy is to promote optimized fetal growth. When asthma is effectively managed, the risk of preterm birth and low birth weight is significantly reduced. This reduction in adverse outcomes allows the developing fetus to progress through gestation with the time and resources needed for optimal growth and development. In contrast, poorly controlled asthma is associated with a heightened risk of preterm birth and low birth weight, which can impede the attainment of these crucial developmental milestones. By prioritizing asthma control, expectant mothers create a nurturing environment that supports the fetus's progression toward a healthier and more robust state [51].

Reduced risk of fetal complications: Adequate asthma control translates to a reduced risk of fetal complications during pregnancy and labor. These complications may include fetal distress and oxygen deprivation, which can occur during labor. Asthma control minimizes the chances of experiencing such complications, contributing to better neonatal outcomes. Reducing the risk of fetal distress and oxygen deprivation is instrumental in fostering a more positive and secure childbirth experience [30].

Enhanced placental function: Maternal respiratory function is intricately linked to placental function, making the optimization of the former pivotal for the well-being of the developing fetus. When asthma is well-controlled, the mother's respiratory system operates efficiently, ensuring the placenta functions optimally. In this context, the placenta delivers a consistent and ample supply of oxygen and essential nutrients to the fetus. This seamless interaction between maternal respiratory health and placental function profoundly impacts fetal development, setting the stage for healthier growth and a more robust foundation for life [52].

Lower risk of neonatal respiratory Issues: Maternal asthma control directly affects the risk of neonatal respiratory issues, such as respiratory distress syndrome (RDS). Ensuring that fetal lung development is supported by optimal maternal respiratory function is vital in reducing the risk of these complications. When maternal asthma is well-managed, the fetus benefits from a more favorable intrauterine environment, where oxygen levels are consistent, fostering the development of healthy and functional lungs. This, in turn, contributes to a decreased likelihood of neonatal respiratory issues and a smoother transition to postnatal life for the newborn. By prioritizing asthma control, expectant mothers are proactive in safeguarding their child's respiratory health, aligning to promote optimal neonatal outcomes [53].

The Role of Healthcare Providers in Optimizing Fetal Health

Education: Educating pregnant women about the impact of asthma on fetal health is crucial. Asthma can affect the fetus's oxygen supply, leading to complications. It's important to inform women about these potential risks. Additionally, guiding safe asthma control strategies during pregnancy is essential. This could include information on avoiding asthma triggers, the proper use of inhalers, and recognizing early signs of an asthma attack [29].

Medication management: Prescribing safe asthma medications and advising on appropriate dosages is a delicate balance. Some asthma medications may potentially harm the fetus, but uncontrolled asthma can also be harmful. Healthcare providers must carefully assess each medication's risks and benefits for the individual patient. This involves considering the asthma's severity and the pregnant woman's needs [54].

Regular monitoring: Routine monitoring of asthma control is critical throughout pregnancy. Maternal lung function, asthma symptoms, and fetal well-being should be assessed regularly. This monitoring allows healthcare providers to make informed decisions about asthma management. For instance, medication adjustments or management strategies may be necessary if asthma worsens during pregnancy [55].

Collaboration: Collaboration with other healthcare professionals, such as obstetricians and neonatologists, is essential. Asthma can affect the mother and the developing fetus, so a coordinated approach is necessary. Obstetricians can provide insights into the impact of pregnancy on asthma and help manage any complications. Neonatologists can be on standby to address potential neonatal issues related to asthma. Working together ensures that any problems are addressed promptly [26].

Individualized care: Every pregnant woman's asthma is unique. Asthma severity can vary, as can the specific triggers that worsen symptoms. The overall health of the individual also plays a role. Tailoring asthma care to the individual's needs is vital. This means developing a customized asthma management plan considering all these factors. What works for one pregnant woman may not suit another, so a personalized approach is critical to successful asthma management during pregnancy [5].

Outcomes of asthma in pregnancy

Maternal Outcomes

Exacerbation-related complications: Poorly controlled asthma during pregnancy can have serious consequences. Exacerbations, or sudden worsening of asthma symptoms, can lead to severe respiratory distress. These exacerbations may require hospitalization and, in rare cases, can be life-threatening for both the pregnant woman and the developing fetus. The decreased oxygen supply during severe asthma attacks can jeopardize fetal well-being. Therefore, healthcare providers must monitor and manage asthma to prevent exacerbations closely and, if they occur, provide immediate medical intervention to safeguard maternal and fetal health [56].

Quality of life: Asthma can significantly impact a pregnant woman's quality of life. Frequent asthma symptoms, exacerbations, and the emotional burden of asthma can create substantial physical and emotional challenges. These challenges can affect daily functioning, overall well-being, and mental health. Managing asthma effectively during pregnancy prevents complications and enhances the pregnant woman's comfort and quality of life. Adequate asthma control can alleviate the physical discomfort and emotional distress associated with asthma symptoms, thus contributing to a better pregnancy experience [57].

Postpartum asthma control: It is essential to recognize that the impact of asthma on a pregnant woman's health does not end with childbirth. Some women may experience changes in asthma control after giving birth. This can be due to hormonal fluctuations, the stress associated with caring for a newborn, or other factors. Healthcare providers should continue to monitor and manage asthma in the postpartum period. Maintaining reasonable asthma control beyond pregnancy is essential for the long-term health and well-being of the mother. Ongoing support and adjustments to the asthma management plan may be necessary to adapt to the individual's changing needs during this period [5].

Fetal and Neonatal Outcomes

Preterm birth: Poorly controlled asthma during pregnancy is associated with an increased risk of preterm birth when a baby is born before 37 weeks of pregnancy is completed. Preterm infants may face various health challenges due to their underdeveloped organs and systems. They are at a higher risk of complications such as RDS, infections, and long-term health issues, including developmental delays and chronic health conditions [58].

Low birth weight: Inadequate asthma control can lead to low birth weight in newborns. Low birth weight is a birth weight of less than 2,500 grams (5.5 pounds). Babies with low birth weight may be at increased risk of health problems in infancy and later life. These health issues include respiratory problems, developmental delays, and an increased likelihood of chronic diseases such as heart disease and diabetes in adulthood [59].

Neonatal respiratory distress: Babies born to mothers with poorly controlled asthma are at risk of experiencing neonatal RDS or other respiratory complications. RDS is a condition in which the baby's lungs have not fully developed, making it difficult for the infant to breathe. This condition often requires neonatal intensive care and interventions like mechanical ventilation and surfactant therapy [60].

Congenital anomalies: While the relationship between maternal asthma and congenital anomalies is an area of ongoing research, some studies have suggested a potential link. It is essential to thoroughly understand and address this connection to ensure the safety of the developing fetus. While the exact causes of congenital anomalies are often complex and multifactorial, poorly controlled asthma during pregnancy may be one of the contributing factors. Healthcare providers must carefully monitor and manage asthma in pregnant women to mitigate potential risks [61].

Long-Term Implications for Both Mother and Child

Maternal health: Poorly controlled asthma during pregnancy can impact the mother's health. It may result in reduced respiratory function and overall well-being. Chronic inflammation and uncontrolled asthma symptoms can lead to persistent airway damage. Severe exacerbations and hospitalizations during pregnancy can also have long-term emotional and psychological effects on the mother, potentially leading to anxiety, depression, and reduced quality of life. Effective asthma management is crucial to preserving the mother's health and mental well-being [5].

Childhood asthma risk: Children born to mothers with asthma are at an increased risk of developing asthma and allergic diseases. The intergenerational impact of maternal asthma highlights the importance of understanding and managing asthma during pregnancy. While genetics plays a role, environmental factors and exposures during pregnancy can influence a child's susceptibility to asthma. Therefore, optimizing asthma control in pregnant women may reduce their offspring's risk of childhood asthma and related health issues [61].

Healthcare utilization: Poorly controlled asthma during pregnancy can lead to increased healthcare utilization for both the mother and child in the years following birth. This includes regular medical appointments, prescription medications, and treatments related to asthma. Managing asthma effectively during pregnancy can help reduce the need for ongoing medical interventions and enhance the mother's and child's well-being [5].

Quality of life: The long-term quality of life for both the mother and child can be influenced by the experiences and management of asthma during pregnancy. Chronic uncontrolled asthma can lead to physical discomfort, emotional distress, and limitations in daily activities. On the other hand, optimizing asthma control can have positive implications for long-term well-being. By effectively managing asthma during pregnancy, the mother can improve her quality of life and provide a healthier start for her child [40].

## Conclusions

In conclusion, this comprehensive review of asthma in pregnancy has shed light on the intricate interplay of factors that influence maternal and fetal health during this crucial period. The prevalence of asthma in pregnant women, combined with the physiological changes induced by pregnancy, underscores the significance of understanding and effectively managing this condition. Proper diagnosis, medication management, personalized asthma action plans, lifestyle modifications, and stress reduction are integral to ensuring optimal asthma control. Healthcare providers are central to this process, offering education, monitoring, and individualized care. The importance of proper asthma management in pregnancy cannot be overstated, as it not only improves maternal and fetal outcomes but also has the potential to impact long-term health, both for the mother and her child. Ongoing research, precision medicine, and a holistic approach to care promise to enhance further outcomes in managing asthma during pregnancy.
